# Factors Predisposing the Response to Lumacaftor/Ivacaftor in People with Cystic Fibrosis

**DOI:** 10.3390/jpm12020252

**Published:** 2022-02-10

**Authors:** Julie Mésinèle, Manon Ruffin, Loïc Guillot, Pierre-Yves Boëlle, Harriet Corvol, on behalf of the French CF Modifier Gene Study Investigators

**Affiliations:** 1 Centre de Recherche Saint-Antoine (CRSA), Inserm, Sorbonne Université, 75012 Paris, France; julie.mesinele@inserm.fr (J.M.); manon.ruffin@inserm.fr (M.R.); 2 Hôpital Saint-Antoine, AP-HP, Institut Pierre Louis d’Epidémiologie et de Santé Publique (iPLESP), Inserm, Sorbonne Université, 75012 Paris, France; pierre-yves.boelle@sorbonne-universite.fr; 3 Service de Pneumologie Pédiatrique, Hôpital Trousseau, AP-HP, Centre de Recherche Saint-Antoine (CRSA), Inserm, Sorbonne Université, 75012 Paris, France

**Keywords:** CFTR modulator therapy, long-term effect, lung function, nutritional status, modifiers genes, solute carrier family genes, cystic fibrosis

## Abstract

Lumacaftor/ivacaftor (LUMA-IVA) therapy is prescribed to people with cystic fibrosis (pwCF) homozygous for the Phe508del-*CFTR* variant to restore CFTR protein function. There is, however, large inter-individual variability in treatment response. Here, we seek to identify clinical and/or genetic factors that may modulate the response to this CFTR modulator therapy. A total of 765 pwCF older than 12 years under LUMA-IVA therapy and with lung function and nutritional measurements available before and after treatment initiation were included. Response to treatment was determined by the change in lung function and nutritional status, from baseline and over the first two years after initiation, and it was assessed by weighted generalized estimating equation models. Gains in lung function and nutritional status were observed after 6 months of treatment (on average 2.11 ± 7.81% for percent predicted FEV_1_ and 0.44 ± 0.77 kg/m^2^ for BMI) and sustained over the 2 years. We observed that the more severe patients gained the most in lung function and nutritional status. While females started with a nutritional status more impaired than males, they had a larger response and regained BMI Z-score values similar to men after 2 years of treatment. We observed no association between variants in solute carrier (*SLC*) genes and the respiratory function response to LUMA-IVA, but the *SLC6A14* rs12839137 variant was associated with the nutritional response. Further investigations, including other genomic regions, will be needed to fully explore the inter-individual variability of the response to LUMA-IVA.

## 1. Introduction

Cystic fibrosis (CF) is a rare, autosomal recessive, life-shortening genetic disease that affects more than 90,000 people worldwide [1]. It is caused by variants in the gene encoding the cystic fibrosis transmembrane conductance regulator (CFTR), a chloride channel expressed in epithelial cells throughout the body. Until one decade ago, only symptomatic treatments were available for people with CF (pwCF). In the last decade, considerable efforts have led to the development of therapies that target the CFTR protein named CFTR modulators. Since 2012, pwCF carrying some *CFTR* gating variants can be treated with ivacaftor, a potentiator therapy which increases the probability of CFTR-channel opening. 

Following this, lumacaftor, a CFTR corrector that improves the processing and trafficking of the Phe508del-CFTR protein, was combined with ivacaftor to treat pwCF homozygous for the Phe508del-*CFTR* variant. A phase 2 trial of this association, lumacaftor-ivacaftor (LUMA-IVA), demonstrated improvements of the lung function, measured by an increase of the percent-predicted forced expiratory volume in one second (ppFEV_1_), and of the nutritional status, evaluated by the body mass index (BMI) [2]. Marketing authorizations were granted in 2015. 

Since then, clinical benefits of LUMA-IVA have been questioned, particularly because of its high cost [3,4]. In addition, phase 3 trials and real-life studies over the first 2 years of treatment demonstrated highly heterogeneous responses in lung function and nutritional status [5,6,7,8,9,10,11]. These studies also highlighted the inter-individual variability of the airway response and the limited tolerance to treatment, with high discontinuation rates ranging from 17.2% to 28.9% [5,7,9]. Predisposing factors for interruption and for the response variability were shown to be baseline lung function, age at treatment initiation and gender [5,7,8,9,10,11]. 

In a previous study, we found that lung response variability to ivacaftor was associated with variants in the *Solute Carrier Family 26 Member 9* (*SLC26A9)* gene in a cohort of French pwCF, confirming results observed in Canadians [12,13]. However, these results were controverted in North American pwCF [14]. Nevertheless, this gene was also shown to be involved in lung function variability [13,15] and to meconium ileus susceptibility in pwCF [16]. Furthermore, other variants in the *SLC* gene family (*SLC6A14* and *SLC9A3*) have been shown to modify several CF phenotypes, suggesting a pleiotropic effect of this gene family of genes [15,16,17,18].

Here, we analysed the evolution of pwCF treated with LUMA-IVA and included the “French CF Gene Modifier Study” to identify clinical and/or genetic factors that may change the response to this CFTR modulator therapy.

## 2. Materials and Methods

### 2.1. Study Design and Patients

The “French CF modifier gene study” is a nationwide observational study of pwCF in France with prospective data collection from participants. Here, we aimed to evaluate the lung and nutritional response to LUMA-IVA over the first two years of treatment. As of 31 January 2021, 4975 pwCF attending one of the 47 French CF centres were included in this national cohort (corresponding to ~70% of all French pwCF) [19].

Since 2001, neonatal CF screening has been underway in France and specialized care for pwCF has been delivered in expert CF centres according to national recommendations [20,21]. Standardized longitudinal data have been prospectively collected for each pwCF, making the current analysis possible. Data collected in electronic or paper medical records over time in the participating CF centres were input in a national database. 

**Figure 1:**
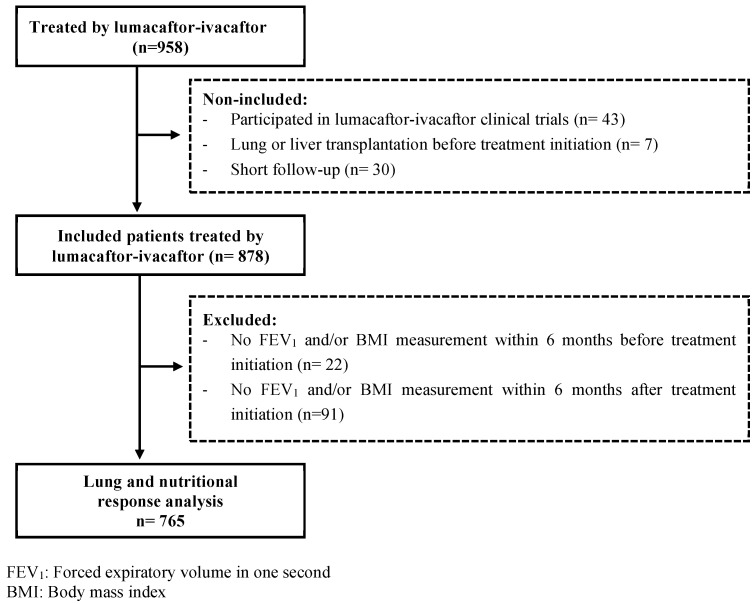
Flowchart of patient selection. Flowchart describing the selection of people with cystic fibrosis included in the analysis of the lung and nutritional response to combined lumacaftor/ivacaftor therapy.

All pwCF older than 12 years, homozygous for the Phe508del-*CFTR* variant and treated by LUMA-IVA out of clinical trials were eligible for inclusion, provided they had at least a 6-month follow-up after treatment initiation and had not received a lung or liver transplant (n = 878) (Figure 1). We excluded pwCF without FEV_1_ and/or BMI values at baseline (n = 22) and/or over the first 6 months after treatment initiation due to missing data (n = 42) or early treatment interruption (n = 49). Finally, 765 pwCF were included in the analyses (Figure 1).

### 2.2. Lung and Nutritional Response to Combined Lumacaftor/Ivacaftor Therapy

According to CF care recommendations, lung function testing including spirometry and anthropometric measurements are performed at every visit in pwCF [21,22]. To assess the lung function, measurements of FEV_1_ were expressed as percent-predicted values using the Global Lung Function Initiative equations [23] or transformed to the Kulich Normalized Mortality Adjusted CF-specific lung phenotype (SaKnorm Z-value) [24,25]. SaKnorm is a quantitative phenotype that allows the direct comparison of lung phenotypes between pwCF and accounts for differential survival. To evaluate the nutritional response, body mass index (BMI) measurements were Z-score transformed according to WHO Child Growth Standards [26]. For each pwCF, SaKnorm Z-value and BMI Z-score baseline values were computed as the measurements’ average over the 6 months prior to the treatment initiation.

We classified the baseline values according to terciles as follows: (i) SaKnorm Z-values from −1.620 to 0.168 (1st tercile), from 0.169 to 0.791 (2nd tercile) and from 0.792 to 2.520 (3rd tercile) for lung response analysis, (ii) BMI Z-score from −4.330 to −1.080 (1st tercile), from −1.079 to −0.307 (2nd tercile) and from −0.306 to 2.890 (3rd tercile) for nutritional response analysis.

Gender, age at treatment initiation, meconium ileus, CF-related diabetes (CFRD), CF-related liver disease (CFLD) and presence of Pseudomonas aeruginosa chronic colonization (*Pa*-CC) were analysed (see Supplementary Information for CFRD, CFLD and *Pa*-CC definitions). Age at treatment initiation was categorized in 3 classes: “≤20” years old, “20–30” years old, and “>30” years old. Changes in FEV_1_ and BMI courses over time according to age at initiation was assessed relative to the “≤20” years old level, indicated as the “reference” level.

### 2.3. Modifiers of Response to Combined Lumacaftor/Ivacaftor Therapy

We analysed single nucleotide polymorphisms (SNPs) located in or near *SLC* genes that had already been shown to be associated with lung response variability to ivacaftor [12,13] and/or several CF phenotypes [15,16,17,18]. Using Kompetitive Allele Specific PCR (LGC Group, Teddington, UK), we genotyped 9 SNPs of the following genes: *SLC26A9* (rs7512462, rs1874361, rs4077468, rs4077469, rs7419153, rs12047830), *SLC9A3* (rs57221529) and *SLC6A14* (rs3788766 and rs12839137).

### 2.4. Statistical Analysis

Descriptive statistics were reported as mean ± standard deviation (SD) or ± standard deviation of mean (SDM) or percentages and 95% confidence intervals (95% CIs), as appropriate. The lung function and nutritional response over the first 2 years of LUMA-IVA therapy were analysed by generalized estimating equation mixed models adapted to longitudinal data. Measurements of SaKnorm Z-value and BMI Z-score were averaged over successive 6-month periods, starting 6 months before treatment initiation (baseline value) and extending over the 2 years following treatment initiation. To account for missing data in patients’ follow-ups, we used generalized estimating equations with weights according to the probability of missingness [27,28]. The missingness model included 4 variables: time since treatment initiation, age at treatment initiation, change in lung function in the previous semester and the baseline lung function value. These criteria included factors associated with treatment discontinuation [7].

For the genetic association study, we applied additive SNP coding, and reference alleles were determined as those with the highest frequency in the European population (http://www.ensembl.org (accessed on 1 June 2021)). SNPs in the chromosome X (*SLC6A14* rs3788766 and rs12839137) were also additively coded (0 or 1 or 2 for women and 0 or 2 for men). Fisher’s exact test was used to test the conformance of the allele frequencies with the Hardy–Weinberg equilibrium.

All analyses were carried out using the R software (version 4.1.0, http://www.R-project.org (accessed on 1 June 2021)) using the package “wgeesel”.

## 3. Results

### 3.1. Study Population

Demographics and baseline characteristics of the eligible pwCF on LUMA-IVA are summarized in Table 1. Distribution is similar across the analysed and the excluded groups by gender, origin, clinical characteristics, lung disease and nutritional severity. Compared to analysed pwCF, excluded pwCF were older (*p*-value < 0.001). Among the 765 pwCF analysed, 14.5% (111 pwCF) discontinued treatment. 

### 3.2. Overall Changes in Lung Function and Nutritional Status with LUMA-IVA

Following the initiation of LUMA-IVA, the lung function and nutritional status improved with gains in SaKnorm Z-value of 0.106 ± 0.015 (*p*-value < 0.0001) and in BMI Z-score of 0.108 ± 0.017 (*p*-value < 0.0001). Improvements were sustained over the 2 years of treatment with change slopes of 0.054 ± 0.010 (*p*-value < 0.0001) for the lung function and of 0.057 ± 0.015 (*p*-value = 0.0001) for the nutritional response. In the first semester, the average absolute gains from baseline were 2.11 ± 0.14% predicted for ppFEV_1_ and 0.44 ± 0.01 kg/m^2^ for BMI (Table S1).

**Figure 2:**
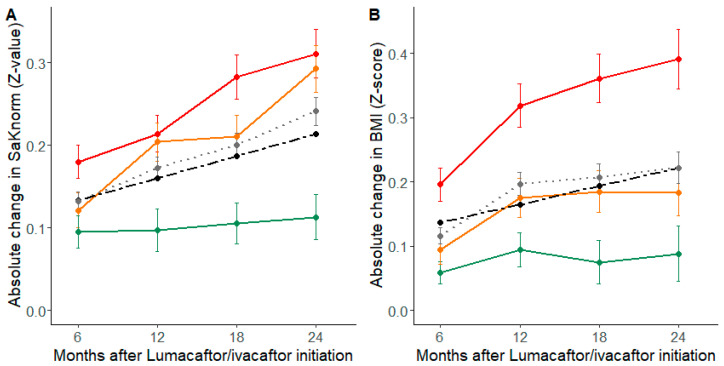
Change according to baseline terciles following initiation of combined lumacaftor/ivacaftor therapy with 95% CIs in (**A**) lung function (SaKnorm Z-value), and (**B**) nutritional status (BMI Z-score), in 765 people with cystic fibrosis (pwCF). For lung response analysis, the baseline SaKnorm (Z-value) terciles were from −1.620 to 0.168 (1st tercile in red), from 0.169 to 0.791 (2nd tercile in orange) and from 0.792 to 2.520 (3rd tercile in green). For nutritional response analysis, the baseline BMI (Z-score) terciles were from −4.330 to −1.080 (1st tercile in red), from −1.079 to −0.307 (2nd tercile in orange) and from −0.306 to 2.890 (3rd tercile in green). The black dotted lines show the model predicted change and the grey dotted lines the overall change in the 765 pwCF.

Treatment response was similar according to age at initiation, presence of meconium ileus, CFRD, CFLD or *Pa*-CC status, but changed by pre-treatment values. Indeed, pwCF with the lowest SaKnorm and BMI baseline values had the greater gains in lung function and nutritional status (Table 2 and Figure 2). 

**Figure 3:**
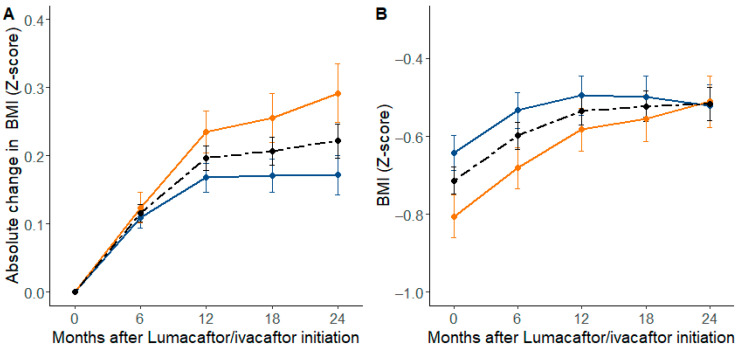
Nutritional status (BMI Z-score) over 2 years following initiation of combined lumacaftor/ivacaftor therapy according to gender with 95% CIs, (**A**) mean of absolute changes from baseline, (**B**) mean of BMI Z-score, in 765 people with cystic fibrosis (pwCF). Men are in blue, women in orange and the dotted lines show the overall change in the 765 pwCF.

At 6 months post-treatment, the pwCF in the baseline 1st tercile had an increased ppFEV_1_ of 2.68 ± 0.25% predicted and of 0.49 ± 0.02 kg/m^2^ for BMI (Table S1). Gender was not associated with lung function response, but females had a higher nutritional response (*p*-value = 0.04) (Table 2 and Figure 3A). At initiation of LUMA-IVA, the baseline nutritional status of women (−0.805 ± 0.992 of BMI Z-score) was more impaired than men (−0.642 ± 0.931 of BMI Z-score). After 2 years of LUMA-IVA, both men and women had a similar nutritional status (−0.511 ± 0.931 and −0.522 ± 0.922 for BMI Z-score for men and women, respectively) (Figure 3B). 

### 3.3. Genetic Analysis

Genotype distributions and results of weighted generalized estimating equation mixed models are shown in Table 3. There was no evidence of association between lung function response to LUMA-IVA and variants of *SLC26A9*, *SLC9A3* or *SLC6A14* genes.

For *SLC26A9* rs7512462 variant and for each additional C-allele, the model estimated a decreased gain in SaKnorm Z-value of −0.006 (±0.017) (*p*-value = 0.71) and in BMI Z-score of −0.023 (±0.023) (*p*-value = 0.32) (Table 3 and Figure 2A). In the case of TT, TC and CC genotypes, over the 6 months post-treatment, the predicted average ppFEV_1_ absolute gains from baseline were 1.95 ± 0.25%, 2.13 ± 0.20% and 2.40 ± 0.32%, respectively (Table S1). 

We observed that only the *SLC6A14* rs12839137 variant was associated with the nutritional response to LUMA-IVA (Table 3). In the first semester, the absolute BMI gains were 0.38 kg/m^2^ (±0.02) and 0.63 kg/m^2^ (±0.03) for the homozygous genotypes GG and AA, respectively, and of 0.47 kg/m^2^ (±0.04) for the GA heterozygous genotypes (Table S1). 

## 4. Discussion

LUMA-IVA was the first promising targeted therapy available for the management of pwCF homozygous for the Phe508del-*CFTR* variant, the most common CF genotype worldwide. Its use in real life has shown a heterogeneous and relatively modest improvement in lung function with limited tolerance. This study analysed clinical factors as well as *solute carrier* variants, located on *SLC26A9*, *SLC9A3* and *SLC6A14* genes, as predictors of lung function and nutritional response to LUMA-IVA.

At 6 months post-treatment, we observed gains of lung function and nutritional status very close to findings of clinical trials that led to the marketing of the therapy [2]. Improvement in lung function severity was sustained over the first 2 years of treatment. The positive slope (+0.054 ± 0.010 of SaKnorm Z-value by year) was reflected in a reduction of ppFEV_1_ decline over the 2 years post-treatment (Table S2), again observed in clinical trials [6]. Among the 878 pwCF who started LUMA-IVA, 19.7% (173 pwCF) interrupted the treatment, similarly to a previously reported rate of discontinuation of 17.2% due to adverse effects [5].

Since 2016 in France, LUMA-IVA treatment can be prescribed to pwCF homozygous for the Phe508del-*CFTR* variant without baseline ppFEV_1_ requirements. We observed that pre-treatment lung function and nutritional status were predictors of treatment response. Indeed, as the severity of pwCF decreased, so did the gain in lung function. The absolute mean change in ppFEV_1_ after 6 months of treatment was +2.68% in the more severe patients (1st SaKnorm Z-value tercile), and +1.89% and +1.77% for patients in the 2nd and 3rd terciles of SaKnorm Z-value, respectively. This pattern was observed in the efficacy analysis, in which improvement in ppFEV_1_ after 6 months of treatment was +3.30% (95% CI: 0.20 to 6.40) in pwCF with baseline ppFEV_1_ < 40% and +2.80% (95% CI: 1.70 to 3.80) in pwCF with baseline ppFEV_1_ ≥ 40% [11]. However, it was also reported that, after 6 months of LUMA-IVA, pwCF with baseline ppFEV_1_ < 40% had a non-significant loss of lung function of −0.40% (−1.90 to 1.10) [8], while those with baseline ppFEV_1_ ≥ 90% had an unchanged lung function of 0.15% [10]. The differences in lung function improvement in these reports and our findings may be due to airways’ response assessment. We used ppFEV_1_ transformed in SaKnorm Z-value to classify pwCF at baseline. This adjustment is an indicator of lung function severity and provides for the direct comparison of pwCF of various ages. We used this specific phenotype to account for age-dependent clinical variables (CFRD, CFLD and *Pa*-CC). In addition, it should be noted that previous studies included fewer than 50 pwCF, whereas the current study analysed 765 pwCF of the “French CF modifier gene study”, which includes more than 85% of all French adult pwCF [29].

Apart from baseline lung function, no demographic or clinical factors were found to be associated with the respiratory response to treatment. Only gender was associated with the nutritional response, with a statistically significant greater change in BMI Z-score in women after LUMA-IVA initiation. This result is in part related to a difference in nutritional severity between men and women before treatment initiation. Indeed, we observed that women gained the same level of nutritional status as men after 2 years of treatment. All studies agree that there is a significant gain in BMI, ranging from 0.21 to 0.96 kg/m^2^, irrespective of the LUMA-IVA dose, lung function severity or treatment duration [2,6,7,8,11]. However, none of these studies showed differences in gender for the nutritional status response. So far, female gender was only associated with a higher discontinuation rate [5].

This study, with a very large cohort of pwCF homozygous for the Phe508del-*CFTR* variant, did not show any evidence of association between *SLC* genes’ family and respiratory response to LUMA-IVA therapy. The *SLC26A9* gene was suggested to modulate the airway response to ivacaftor in pwCF who harbour at least one of the targeted *CFTR* gating variants [12,13,14]. Moreover, ex vivo experiments with nasal epithelial cells from pwCF showed that the *SLC26A9* rs7512462 variant was associated with CFTR function in response to LUMA-IVA [30]. However, we observed no association of this variant with either the respiratory or the nutritional response to LUMA-IVA. Among the variants analysed, only the *SLC6A14* rs12839137 variant was associated with the nutritional response. Interestingly, two studies in Finnish and French showed that several *SLC6A14* variants were associated with obesity [31,32]. As the amino acid transporter SLC6A14 could modulate tryptophan availability for serotonin synthesis, authors hypothesized that these variants might affect perception of hunger and satiety. Miranda et al. also showed that other *SLC6A14* variants were associated with food intake in children at 7–8 years of age [33]. Finally, an in vitro study highlighted that the obesity-associated rs2011162 variant, located in the 3′UTR of the *SLC6A14* gene, reduces the expression of the transporter and that *Slc6a14*^−/−^ mice develop obesity, fatty liver and metabolic syndrome when fed with a high-fat diet [34]. These findings support the hypothesis of the involvement of *SLC6A14* in the regulation of the nutritional status in pwCF.

Our study has several limitations. First, missing information led to the exclusion of pwCF from the analysis of FEV_1_ and BMI change. According to French CF care recommendations, lung function and anthropometric measurements are measured every quarter in pwCF [21], and, following recommendations of the French CF Learning Society, each pwCF who initiates LUMA-IVA has systematic visits at treatment initiation and at 1, 3, 6 and 12 months after initiation (with clinical assessment and a pulmonary function test) [9]. Missing data could be due to the collection date from patients’ paper and electronic medical records. However, we did not observe any clinical differences between included and excluded patients. Secondly, we observed a 14.5% treatment interruption among the 765 pwCF analysed, of which 10.81% (n = 12) could be attributed to a lack of observed benefits by the patient and/or physician. It was also shown that the risk of discontinuation increased with age and severity of lung function [7]. However, to account for this selection bias and shorter follow-up times, we used weighted statistical models to estimate the probability of missing data, as a function of age at treatment initiation, change in lung function at the prior semester and the baseline lung function value.

## 5. Conclusions

LUMA-IVA shows modest benefits in terms of lung function and nutritional status in pwCF. However, benefits in quality of life should not be overlooked, as well as the reduction of respiratory exacerbations, which have a major role in the CF management. We showed that variants located in the *SLC* gene family (*SLC26A9*, *SLC9A3* and *SLC6A14)* were not predictors of the airway treatment response and further investigations, including of other genomic regions, are needed to better explain inter-individual variability. Nevertheless, we observed that the variant rs12839137 of the *SLC6A14* gene was associated with the nutritional response, which will be important to further explore. With the development of the next-generation CFTR modulators, such as the elexacaftor/ivacaftor/tezacaftor tritherapy, identifying genetic modifiers involved in the response to treatment is an opportunity to achieve predictive and personalized medicine in CF.

## Figures and Tables

**Table 1:** Demographics and baseline (prior to combined lumacaftor/ivacaftor therapy) characteristics of the 878 eligible people with cystic fibrosis.

	Patients Analysed n = 765	Patients Excluded n = 113	*p*-Value
Male, % (n)	56.21% (430)	46.02% (52)	0.042
Caucasian origin, % (n)	97.51% (745)	97.35% (110)	0.915
Age at treatment initiation (years), mean ± SD	22.2 ± 9.0	25.2 ± 9.4	<0.001
Age at treatment initiation (years), % (n)			
≤20	50.07% (383)	31.86% (36)	0.001
20–30	28.37% (217)	38.05% (43)	
>30	21.57% (165)	30.09% (34)	
Presence of meconium ileus % (n)	16.57% (118)	25.47% (27)	0.025
Presence of CFRD *, % (n)	25.93% (195)	31.13% (33)	0.256
Presence of CFLD *, % (n)	36.04% (275)	45.54% (51)	0.052
Presence of *Pa*-CC *, % (n)	38.56% (295)	44.25% (50)	0.248
Lung disease severity ^‡^ (SaKnorm Z-value), mean ± SD	0.447 ± 0.702	0.457 ± 0.710	0.942
Nutritional severity ^†^ (BMI Z-score), mean ± SD	−0.677 ± 0.932	−0.807 ± 1.060	0.072

* Before treatment initiation; ^‡^ Over the past 3 years before treatment initiation, forced expiratory volume in one second (FEV_1_) measurements were expressed as Kulich Normalized Mortality Adjusted CF-specific lung phenotype (SaKnorm Z-value) (1,2); ^†^ Over the past 3 years before treatment initiation, body mass index (BMI) measurements were Z-score transformed according to WHO Child Growth Standards (3). Among the 878 eligible patients, 1 had missing data for ethnicity, 60 for meconium ileus, 20 for CFRD and 3 for CFLD. Abbreviations: CFRD: cystic fibrosis-related diabetes, CFLD: cystic fibrosis liver disease, *Pa*-CC: *Pseudomonas aeruginosa* chronic colonization.

**Table 2:** Lung function and nutritional response of combined lumacaftor/ivacaftor therapy, according to clinical and demographic characteristics in 765 people with cystic fibrosis.

	Change in SaKnorm(Z-Value) ^‡^ ± SD	*p*-Value	Change in BMI(Z-Score) ^†^ ± SD	*p*-Value
Female	−0.015 ± 0.023	0.5203	0.069 ± 0.034	0.0415
Age at initiation (Years)				
≤20	Reference	Reference	Reference	Reference
20–30	0.011 ± 0.026	0.6748	0.006 ± 0.037	0.8705
>30	−0.068 ± 0.023	0.0029	−0.048 ± 0.038	0.2047
Presence of meconium ileus	−0.019 ± 0.031	0.5401	0.010 ± 0.048	0.8407
Presence of CFRD *	−0.003 ± 0.023	0.9066	−0.011 ± 0.039	0.7698
Presence of CFLD *	−0.004 ± 0.024	0.8839	0.003 ± 0.036	0.9414
Presence of *Pa*-CC *	0.018 ± 0.023	0.4380	−0.022 ± 0.034	0.5116
Baseline				
3rd tercile	Reference	Reference	Reference	Reference
2nd tercile	0.105 ± 0.027	0.0001	0.080 ± 0.036	0.0273
1st tercile	0.145 ± 0.028	<0.0001	0.237 ± 0.040	<0.0001

^‡^ Forced expiratory volume in one second (FEV_1_) measurements were expressed as Kulich Normalized Mortality Adjusted CF-specific lung phenotype (SaKnorm Z-value) (1,2); ^†^ Body mass index (BMI) measurements were Z-score transformed according to WHO Child Growth Standards (3); * Before initiation of lumacaftor/ivacaftor combination therapy. *Abbreviations:* CFRD: cystic fibrosis-related diabetes, CFLD: cystic fibrosis liver disease, *Pa*-CC: *Pseudomonas aeruginosa* chronic colonization.

**Table 3:** Lung function and nutritional response of combined lumacaftor/ivacaftor therapy, according to *SLC26A9*, *SLC9A3* and *SLC6A14* variants, in 765 people with cystic fibrosis.

	Position ^£^	Alleles *	MAF EUR	MAF Cohort	HWE **	Change in SaKnorm Z-Value ^‡^ ± SD	*p*-Value	Change in BMI Z-Score ^†^ ± SD	*p*-Value
*SLC26A9*									
rs1874361	1:205939058	**A**/C	0.48	0.46	0.419	−0.006 ± 0.017	0.7338	0.009 ± 0.022	0.6919
rs4077468	1:205945629	A/**G**	0.41	0.41	0.406	−0.011 ± 0.016	0.4976	−0.019 ± 0.023	0.4178
rs4077469	1:205945757	C/**T**	0.41	0.41	0.496	−0.013 ± 0.016	0.4177	−0.023 ± 0.023	0.3350
rs7419153	1:205948181	G/**A**	0.38	0.41	0.033	0.011 ± 0.017	0.4894	0.015 ± 0.024	0.5456
rs7512462	1:205930467	T/**C**	0.41	0.41	0.451	−0.006 ± 0.017	0.7102	−0.023 ± 0.023	0.3166
rs12047830	1:205947571	**G**/A	0.49	0.47	0.501	−0.011 ± 0.017	0.5290	−0.017 ± 0.024	0.4936
*SLC9A3*									
rs57221529	5:586509509	A/**G**	0.21	0.20	0.735	0.029 ± 0.019	0.1256	0.022 ± 0.029	0.4564
*SLC6A14*									
rs3788766	X:116435671435671	**G**/A	0.36	0.37	0.388	0.000 ± 0.014	0.9719	0.008 ± 0.019	0.6797
rs12839137	X:116434382	G/**A**	0.22	0.21	0.862	0.000 ± 0.017	0.9852	0.045 ± 0.020	0.0276

^£^ Physical position according to Ensembl GRCh38 (www.ensembl.org (accessed on 1 June 2021)); * Minor allele in European Population are in bold; ** Hardy–Weinberg equilibrium (HWE) *p*-values were computed by Fisher’s exact test and among women for SLC6A14; ^‡^ Forced expiratory volume in one second (FEV1) measurements were expressed as Kulich Normalized Mortality Adjusted CF-specific lung phenotype (SaKnorm Z-value) (1,2); ^†^ Body mass index (BMI) measurements were Z-score transformed according to WHO Child Growth Standards (1). MAF: minor allele frequency.

## Data Availability

The data presented in this study are available on request from the corresponding author. The data are not publicly available due to ethical restrictions.

## Supplementary Materials

The following supporting information can be downloaded at: https://www.mdpi.com/article/10.3390/jpm12020252/s1, Supplementary Information (phenotype definition, Analysis). Table S1: Mean of lung and nutritional response to combined lumacaftor/ivacaftor therapy within 6 months following initiation, in 765 people with cystic fibrosis. Table S2: Lung function decline in people with cystic fibrosis according to lumacaftor/ivacaftor status. Reference [35] is cited in the Supplementary Materials.
