# Supplementary material for: Factors Predisposing the Response to Lumacaftor/Ivacaftor in People with Cystic Fibrosis
Source: J Pers Med. 2022 Feb 10;12(2):252. doi: 10.3390/jpm12020252 (PMC8876860; doi:10.3390/jpm12020252)
Supplement: Supplementary file 1 [file jpm-12-00252-s001.zip › jpm-1536016-supplementary.pdf]

FACTORS PREDISPOSING THE RESPONSE TO LUMACAFTOR/IVACAFTOR IN PEOPLE WITH CYSTIC FIBROSIS

SUPPLEMENTARY MATERIAL

1 Phenotype Definition

1.1 Cystic Fibrosis Related Diabetes (CFRD) Definition

CF French guidelines [1] recommend that people with CF (pwCF) aged 10 years and older be tested every year for CFRD with an oral glucose tolerance test (OGTT). CFRD is diagnosed with 2 h oral glucose tolerance test values of more than or equal to 11.1 mmol/L (200 mg/dL). Here, CFRD was defined as *"present"* when it occurred before age at initiation of the lumacaftor/ivacaftor (LUMA-IVA) therapy.

1.2 Cystic Fibrosis Related Liver Disease (CFLD) Definition

CFLD was defined as previously reported according to the European best-practice guidelines [2,3]:

- At least two of the following characteristics were present: (1) abnormal physical examination, including hepatomegaly and/or splenomegaly; (2) abnormalities in liver function tests defined as an increase in transaminase (alanine aminotransferase and/or aspartate aminotransferase) and/or gamma-glutamyl transpeptidase levels above the upper normal limits; or (3) ultrasonographic (US) evidence of liver involvement (heterogeneous echogenicity, irregular margins, or nodularity), portal hypertension (splenomegaly, increased thickness of the lesser

omentum, spontaneous splenorenal anastomosis, large collateral veins, or ascites), or biliary abnormalities (bile duct dilatation).

- When patients had severe CFLD defined when at least one of the following characteristics were present: (1) cirrhosis diagnosed either by US, computed tomography, and/or magnetic resonance imaging; (2) portal hypertension (splenomegaly, hypersplenism (platelets <150,000 10^9/L and white blood cells <3000 10^9/L); (3) spontaneous portosystemic shunts on US; or (4) oesophageal varices.

CFLD was defined as *"present"* when it occurred before age at initiation of the LUMA-IVA therapy.

1.3 Pseudomonas Aeruginosa Chronic Colonisation (Pa-CC) Definition

Microbiological analyses of sputum samples were conducted quarterly in expert laboratories affiliated with CF centres in all pwCF, according to French CF care recommendations [1]. Physicians determined the date of *Pa*-CC according to the standardised definition: at least three positive samples at least 1 month apart over a 6-month period [1]. Age at *Pa*-CC was the age at the third isolation. Here, *Pa*-CC was defined as *"present"* when it occurred before age at initiation of the LUMA-IVA therapy.

2 Analysis

Impact of LUMA-IVA on lung function decline:

We used linear mixed model regression to compute the change in $ppFEV_1$ according to LUMA-IVA status. For each 765 pwCF included, all longitudinal $ppFEV_1$ data 3 years before and 2 years after LUMA-IVA initiation were included in the model. We described individual trajectories of $ppFEV_1$ by a segmented regression with changes in slope at the age at LUMA-IVA initiation and estimated the average change per year in the periods before and after LUMA-IVA initiation. More precisely, the model was:

$$ppFEV_1\ (a) = b_0 + b_1\ age + b_2\ (age - age_{LUMA\text{-}IVA})_+ + b_3\ (treatment) + \varepsilon;$$

Where b_1 was the slope corresponding to the natural decline of $ppFEV_1$ with age, b_2 was the additional slope after LUMA-IVA initiation, $()_+$ was the positive part function, *treatment* a binary variable (coded 0 if $ppFEV_1$ measurement was before and coded if 1 $ppFEV_1$ measurement was after LUMA-IVA initiation) and ε was a normally distributed error. We used random intercepts and slopes to capture the heterogeneity in decline in $ppFEV_1$.

Among the765 pwCF, we observed the average annual rate of change in $ppFEV_1$ was -1.86 %/year (95% CI: -2.04 to -1.68) in pwCF before LUMA-IVA initiation (Table S2). The decrease was slower after LUMA-IVA initiation with an annual rate of change in $ppFEV_1$ of -0.81/year (95% CI: -1.22 to -0.40) (Table S2).

3 Supplementary Tables

Table S1. Mean of lung and nutritional response to combined lumacaftor/ivacaftor therapy within 6 months following initiation, in 765 people with cystic fibrosis.

	Change in FEV_1 (% Pred)* Mean ± SDM	Change in SaKnorm (Z-Value)‡ Mean ± SDM	Change in BMI Mean ± SDM	Change in BMI (Z-Score)† Mean ± SDM
**Overall**	2.11 ± 0.14	0.13 ± 0.01	0.44 ± 0.01	0.12 ± 0.01
**Gender**				
Male	2.01 ± 0.18	0.13 ± 0.01	0.44 ± 0.02	0.11 ± 0.01
Female	2.24 ± 0.22	0.13 ± 0.01	0.44 ± 0.02	0.12 ± 0.01
**Baseline**				
1st tercile	2.68 ± 0.25	0.18 ± 0.01	0.49 ± 0.02	0.20 ± 0.01
2nd tercile	1.89 ± 0.26	0.12 ± 0.01	0.43 ± 0.03	0.09 ± 0.01
3rd tercile	1.77 ± 0.23	0.10 ± 0.01	0.40 ± 0.02	0.06 ± 0.01
***SLC26A9*, rs7512462**				
TT	1.95 ± 0.25	0.12 ± 0.01	0.46 ± 0.02	0.13 ± 0.01
TC	2.13 ± 0.20	0.13 ± 0.01	0.42 ± 0.02	0.11 ± 0.01
CC	2.40 ± 0.32	0.16 ± 0.01	0.42 ± 0.03	0.10 ± 0.01
***SLC6A14*, rs12839137**				
GG	1.86 ± 0.18	0.12 ± 0.01	0.38 ± 0.02	0.09 ± 0.01
GA	2.26 ± 0.35	0.13 ± 0.01	0.47 ± 0.04	0.13 ± 0.02
AA	2.66 ± 0.35	0.16 ± 0.01	0.63 ± 0.03	0.18 ± 0.01

* Forced expiratory volume in one second (FEV_1) measurements were expressed as percent-predicted values ($ppFEV_1$) using the Global Lung Function Initiative equations [4]; ‡Forced expiratory volume in one second (FEV_1) measurements were expressed as Kulich Normalized Mortality Adjusted CF-specific lung phenotype (SaKnorm Z-value) [5,6], †Body mass index (BMI) measurements were Z-score transformed according to WHO Child Growth Standards [7]. *Abbreviation*: DSM: standard deviation of mean.

Table S2. Lung function decline in people with cystic fibrosis according to lumacaftor/ivacaftor status.

Parameter	Estimate (95% CI)	P-Value **
$ppFEV_1$ * change rate before LUMA-IVA initiation (%/ year)	-1.86 (-2.04; -1.68)	<0.0001
Additional change rate after LUMA-IVA initiation (%/ year)	1.06 (0.60; 1.51)	<0.0001

* Forced expiratory volume in one second (FEV_1) measurements were expressed as percent-predicted values ($ppFEV_1$) using the Global Lung Function Initiative equations [4], allowing adjustment of the model on age, sex, and height; ** For comparison to 0. *Abbreviation:* LUMA-IVA: lumacaftor/ivacaftor therapy.

4 References

1. HAS. Mucoviscidose: Protocole national de diagnostic et de soins pour une maladie rare. Guide - Affection de longue durée. 2017.
2. Debray D, Kelly D, Houwen R, Strandvik B, Colombo C. Best practice guidance for the diagnosis and management of cystic fibrosis-associated liver disease. J Cyst Fibros. 2011;10 Suppl 2:S29-36.
3. Boelle PY, Debray D, Guillot L, Clement A, Corvol H, French CFMGSI. Cystic Fibrosis Liver Disease: Outcomes and Risk Factors in a Large Cohort of French Patients. Hepatology. 2019;69(4):1648-56.
4. Quanjer PH, Stanojevic S, Cole TJ, Baur X, Hall GL, Culver BH, et al. Multi-ethnic reference values for spirometry for the 3-95-yr age range: the global lung function 2012 equations. The European respiratory journal. 2012;40(6):1324-43.
5. Kulich M, Rosenfeld M, Campbell J, Kronmal R, Gibson RL, Goss CH, et al. Disease-specific reference equations for lung function in patients with cystic fibrosis. Am J Respir Crit Care Med. 2005;172(7):885-91.
6. Taylor C, Commander CW, Collaco JM, Strug LJ, Li W, Wright FA, et al. A novel lung disease phenotype adjusted for mortality attrition for cystic fibrosis Genetic modifier studies. Pediatr Pulmonol. 2011;46(9):857-69.
7. de Onis M, Onyango AW, Borghi E, Siyam A, Nishida C, Siekmann J. Development of a WHO growth reference for school-aged children and adolescents. Bull World Health Organ. 2007;85(9):660-7.
